# Template Stripping Method-Based Au Nanoarray for Surface-Enhanced Raman Scattering Detection of Antiepileptic Drug

**DOI:** 10.3390/mi11100936

**Published:** 2020-10-14

**Authors:** Tatsuro Endo, Hirotaka Yamada, Kenji Yamada

**Affiliations:** 1 Graduate School of Engineering, Osaka Prefecture University, 1-1 Gakuen-cho, Naka-ku, Sakai-shi, Osaka 599-8531, Japan; sxb02146@edu.osakafu-u.ac.jp; 2 JST PRESTO, 4-1-8 Honcho, Kawaguchi, Saitama 332-0012, Japan; 3 Department of Electronics & Computer Engineering, Hiroshima Institute of Technology, 2-1-1 Miyake, Saeki-ku, Hiroshima 731-5193, Japan; k.yamada.7a@cc.it-hiroshima.ac.jp

**Keywords:** plasmonic crystal, template stripping method, surface-enhanced Raman scattering, phenobarbital, antiepileptic drug

## Abstract

Surface-enhanced Raman scattering (SERS) is a potential candidate for highly sensitive detection of target molecules. A SERS active substrate with a noble metal nanostructure is required for this. However, a SERS active substrate requires complicated fabrication procedures. This in turn makes it difficult to fabricate highly sensitive SERS active substrates with high reproducibility. To overcome this difficulty, a plasmonic crystal (PC) with periodic noble metal nanostructures was fabricated via the template-stripping method using a polymer-based template. Using SERS active substrates, SERS was successfully achieved using the PC by detecting low concentrations of phenobarbital which is an antiepileptic drug using a commercially available portable Raman module. The PC can be fabricated by demolding the deposited gold layer from a polymer-based template. This method is rapid, economic, and has high reproducibility. SERS can be achieved easily using this PC for a wide variety of applications such as medical, pharmaceutical, and environmental protection.

## 1. Introduction

The comprehension of biological functions like DNA replication, RNA and protein synthesis, and metabolism requires the detection and determination of target molecules in body fluids, cells, and tissues [1]. Target molecules can be detected and determined using liquid chromatography [2], electrophoresis [3], mass spectroscopy [4], fluorescent microscopy [5], and biochemical analytical tools, such as an enzyme-linked immunosorbent assay [6]. These instruments and methods are critical tools for understanding biological functions. However, these commonly used instruments and methods pose difficulties in the detection of low molecular weight compounds, such as target molecules. Several methods have been specifically designed to detect low molecular weight compounds with high sensitivity. Kranz et al. reported the thermal shift assay to detect low molecular weight compounds [7]. Using this assay, fractional binding between target molecules and recognition elements which specifically bind to the target molecules, such as antibody and probe DNA can be detected with high sensitivity. From the viewpoint of instrument and method development, Osypova et al. reported the quartz crystal microbalance and aptamer for the detection of low molecular weight compounds [8]. These previously reported instruments and methods enable the detection of low molecular weight compounds cost-effectively, and with high sensitivity. However, the requirements of recognition elements such as aptamer and antibody are still unimproved.

To overcome the requirement of recognition elements, we focused on Raman spectroscopy [9,10,11]. Raman spectroscopy is one of the vibrational spectroscopic techniques used to provide information on molecular vibrations and crystal structures of samples [12]. The spectral specificity of Raman spectroscopy has been recognized as a powerful tool for identifying target molecules. The detection of low molecular weight compounds can be performed using Raman spectroscopy, specifically without recognition elements by monitoring Raman spectra. Raman spectroscopy helps in the detection of low molecular weight compounds with high sensitivity. Conventional Raman spectroscopy monitors only weak Raman scattering signals. To overcome this drawback, surface-enhanced Raman scattering (SERS) to enhance the Raman scattering signals has been studied theoretically and experimentally [13].

SERS enables the enhancement of Raman scattering signals that cannot be measured using conventional Raman spectroscopy because of the low concentrations of target molecules. SERS can be realized only if the target molecules are positioned close to the surface of metal on rough metal surfaces or nanostructures such as nanoparticles [14,15,16]. When the target molecules are absorbed on the rough metal surface or nanostructures, the Raman spectra are enhanced, owing to the excitation of localized surface plasmon resonance (LSPR) [17]. SERS-active substrates with metal nanostructures have been studied to perform SERS [18]. Freeman et al. reported a metal colloid monolayer assembled substrate for SERS [19]. Using this substrate, LSPR can significantly enhance the Raman signal. Chung et al. reported the fabrication of metal (silver or gold) nanostructures using anodized aluminum oxide as the template [20]. Using this substrate, the dengue virus could be detected with high sensitivity. SERS active substrates thus have considerable potential for detecting low molecular weight compounds and can help understand biological functions. However, the reproducibility of the fabricated SERS active substrate is a significant challenge. The fabrication of a SERS active substrate using the template stripping method was the objective of this study.

The template stripping method was originally developed for the preparation of ultra-flat gold surfaces [21,22,23]. Using this method, a structure reflected from the template could be fabricated rapidly, easily, and cost-effectively. From the previous reports, SERS active substrate using nanoimprint lithography (NIL) had been fabricated [24,25]. However, for the fabrication of SERS active substrate, a solid substrate such as a silicon had been used as a template. Using a solid substrate as a template, reproducibility will be affected by the damage of the template. On the other hand, we tried to use the polymer-based template for the fabrication of SERS active substrate. By using a polymer-based template for fabrication of SERS active substrate, SERS active substrate can be fabricated with high reproducibility by changing the polymer-based template. We have fabricated polymer-based nanostructures using NIL to develop nanophotonics-based biosensors that operate in the visible region [26,27,28,29,30,31]. The surface energy of a polymer is generally low. Hence, when a metal layer was deposited onto a polymer surface, the metal layer could be peeled off easily. Polymer-based nanostructures can be used as templates to transfer metal nanostructures. We successfully fabricated periodic metal nanostructures called “plasmonic crystals (PCs)” for the excitation of LSPR. PCs were also used for the highly sensitive detection of an antigen-antibody reaction [32]. By using this PCs, arranged nanostructures enable the observation of lattice resonance [33]. In addition, lattice resonance led to a drastic change in the LSPR optical characteristics attributed to the change in effective refractive index due to the antigen-antibody reaction. The PCs were thus successfully used as SERS-active substrates. The PCs were used to detect phenobarbital. Phenobarbital is a seizure medicine for epilepsy [34]. The evaluation of drug efficacy can be performed by high-sensitivity detection of phenobarbital in body fluids, such as tears using PC [35].

## 2. Materials and Methods

### 2.1. Reagents

To fabricate the polymer-based template, a cycloolefin polymer (COP) film (thickness: 100 µm) was obtained from Zeon Corp. (Tokyo, Japan). To deposit a gold layer onto the template surface, gold beads (99.99% (*w*/*w*)) were purchased from Tanaka Kikinzoku Kogyo K. K. (Tokyo, Japan). Thermosetting epoxy resin (EPO-TEK^®^ 377) purchased from Epoxy Technology (Billerica, MA, USA) was used to bond the gold layer.

### 2.2. Apparatus

A thermal evaporator (SVC-700TM/700-2) from Sanyu Electron (Tokyo, Japan) was used to deposit a gold layer onto the polymer-based template. The base pressure was monitored using an analog ionization vacuum gauge (ULVAC GI-TL3, Kanagawa, Japan). The rate of increase in thickness was monitored using a deposition monitor (INFICON XTM2, Kanagawa, Japan). To monitor the LSPR optical characteristics, a handy-type spectrometer (Ocean Optics USB-4000-UV-VIS, wavelength range: 200–1100 nm, Dunedin, USA), a tungsten halogen light source (Ocean Optics LS-1, wavelength range: 250–800 nm), and an optical fiber probe bundle (Ocean Optics R-400-7 UV/VIS, fiber core diameter: 400 µm, wavelength range: 300–1100 nm) were used. A commercially available portable Raman module (C12710, laser wavelength: 785 nm, power: 3 mW or 50 mW) was obtained from Hamamatsu Photonics K.K. (Shizuoka, Japan) and used for SERS.

### 2.3. Fabrication of Polymer-Based Template

**Figure 1:**
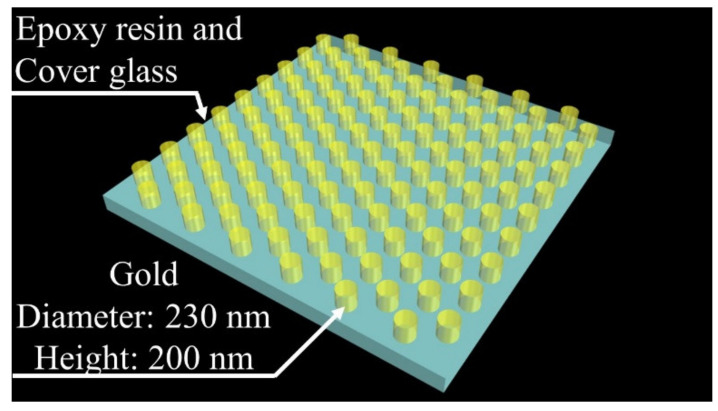
Schematic illustration of configuration of polymer-based template.

A schematic illustration of the configuration of the polymer-based template is shown in Figure 1. In this study, a triangular configured hole array (hole diameter and distance: 230 nm, hole depth: 200 nm) was fabricated using an NIL instrument (X-300, SCIVAX Corp. (Kanagawa, Japan)) as a model case from the previously fabricated PC [32]. From our previous study, changing the thickness and diameter of gold disks will be affected the LSPR optical characteristics. After fabricating the polymer-based template, the PC for SERS was fabricated.

### 2.4. Fabrication of Template Stripping Method-Based PC Using Polymer-Based Template

**Figure 2:**
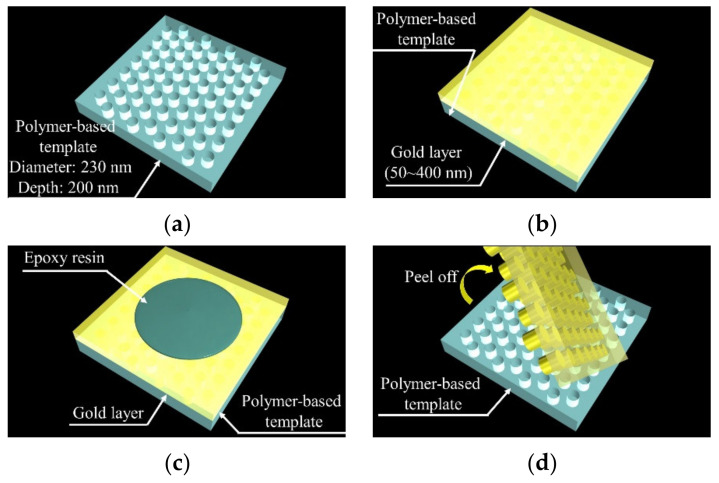
Fabrication procedure of plasmonic crystal (PC) using template stripping method. (**a**) Fabrication of polymer-based template using NIL. (**b**) Deposition of gold layers. (**c**) Bonding of gold layers to cover grass. (**d**) Mechanical peeling of polymer-based template.

The fabrication procedure of PCs for SERS is shown in Figure 2. The polymer-based template—fabricated using NIL—was used to fabricate the PC. Using this design of polymer-based template, we developed a nanophotonic-based biosensor based on a photonic crystal (PhC) [26,27,28,29,30,31]. In addition, using this polymer-based template mold, the metallic layer deposited can be peeled off easily.

To fabricate the PCs, gold layers with different thicknesses (thickness: (50, 100, 200, and 400 nm) were deposited onto the polymer-based template surface (deposition rate: 0.1 nm/s) (Figure 2a) for evaluation of the usability of template stripping method. After deposition (Figure 2b), the gold layers were bonded with cover glass (thickness: 100 µm) using thermosetting epoxy resin at 120 °C for 1 h (Figure 2c). The polymer-based template was then mechanically peeled off from the gold layer (Figure 2d). Through the peeling procedure, the periodically arranged pillar array gold nanostructure was transferred to the cover glass via the thermosetting epoxy resin. After fabricating the PC, its surface morphology was observed using scanning electron microscopy (SEM). The LSPR optical characteristics were also monitored using a handy-type spectrometer.

### 2.5. Simulation Analysis of Electric Field Distribution Using PC

For SERS using PCs, LSPR should be excited with a light source (wavelength: 785 nm). The excitation of LSPR was confirmed using simulation analysis with finite-difference time-domain (FDTD) simulation software (FDTD solution, Lumerical Inc. (Vancouver, BC, Canada)). Using the simulation software, comparison of LSPR excitation efficiency at different wavelengths (532, 635, 650, and 785 nm, which are the wavelengths of commercially available lasers) was performed by monitoring the electric field distribution two-dimensionally. For monitoring of electric field distribution, the plane wave at different wavelength were irradiated to the PC from the perpendicular direction. Furthermore, the cross-sectional electric field distribution was monitored. In addition, using simulation software, LSPR peaks were monitored using a similar configuration of our PC.

### 2.6. SERS Using Template Stripping Method-Based PC by Detection of Phenobarbital

After the fabrication of the PC, SERS for the detection of phenobarbital was carried out using a portable Raman module. The PC surface was irradiated with a laser. The Raman spectrum was then monitored. For SERS using the PC, different concentrations of phenobarbital aqueous solution (0.01–10 µM) or PBS buffer (pH 7.4) were introduced onto the PC surface (20 µL). After introducing the phenobarbital solution or PBS buffer, it was dried. The Raman spectrum of phenobarbital was monitored (time for signal acquisition: 10 sec). In this study, 50 mW laser power was used for SERS application.

## 3. Results and Discussion

### 3.1. Fabrication of Template Stripping Method-Based PC Using Polymer-Based Template

**Figure 3:**
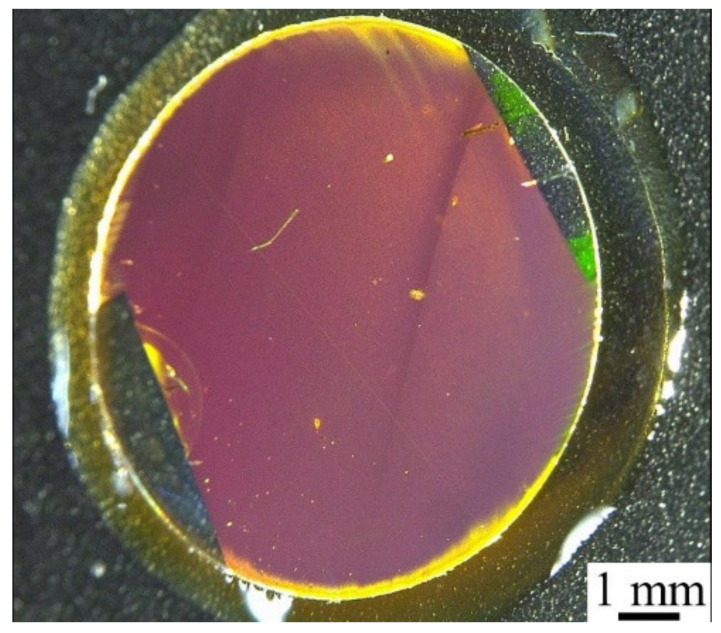
Photograph of PC fabricated using template stripping method.

A photograph of the PC (thickness: 200 nm) is shown in Figure 3. A specific color (purple) due to the LSPR could be observed by the naked eye when the template stripping method was used. A PC with a large area (diameter: 1 cm) was successfully fabricated rapidly and cost-effectively with high reproducibility.

**Figure 4:**
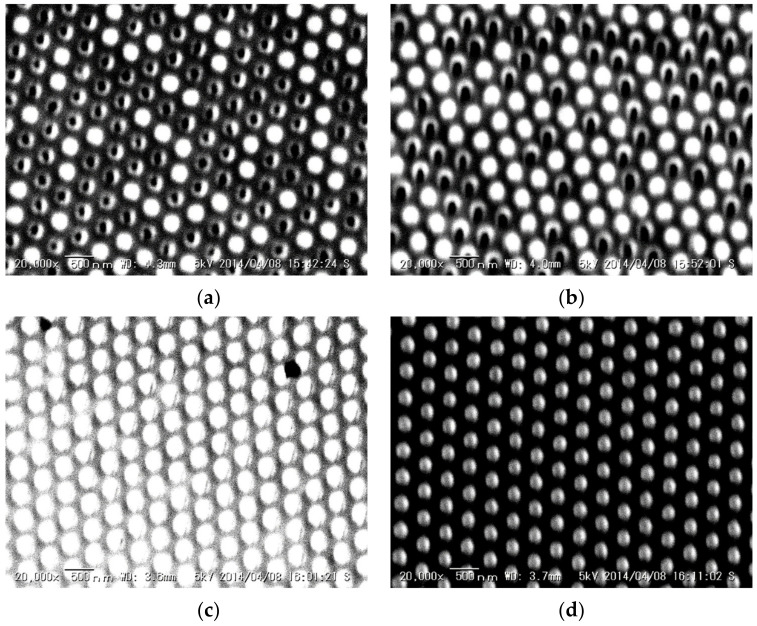
SEM images of PCs fabricated using different thicknesses of the gold layer. (**a**) 50 nm. (**b**) 100 nm. (**c**) 200 nm. (**d**) 400 nm.

SEM images of the PCs, with different thicknesses of the gold layer, are shown in Figure 4. From these SEM images, it is clear that the gold layer thickness affects the transfer efficiency. In this study, the hole depth of the polymer-based template was 200 nm. If the thickness of the gold layer (50 and 100 nm) was lower than the hole depth, defects could be observed. These defects were expected to be due to the differences in the penetration depths of the thermosetting epoxy resin into the hole because of its viscosity. If the thickness was similar to (200 nm) or higher (400 nm) than the hole depth, the reflected gold nanostructure could be fabricated clearly. In the case of a higher thickness of the gold layer (400 nm), the bulk metal color could be observed. According to our previous study, the gold nanostructure should be separated independently to excite the LSPR [32]. The LSPR could not be excited when the gold nanostructures were contacted by the gold layer, as in the case of higher thicknesses of the gold layer. From these results, in this study, PC was fabricated at 200 nm of gold layer for SERS application. By using this fabrication condition and gold layer thickness (200 nm), this PC can be fabricated rapidly with high reproducibility (defect formation ratio: 0.67%) by using template stripping method. In addition, using this fabrication procedure, polymer-based template can be reused for several times because the gold layer can be peeled off from the polymer-based template. However, the polymer-based template was used for over four times, defect formation ratio was drastically increased by the deflection due to the mechanical peeling.

### 3.2. Simulation Analysis of Electric Field Distribution Using PC

**Figure 5:**
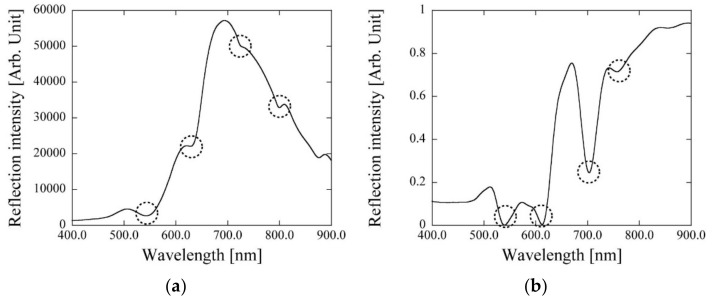
Localized surface plasmon resonance (LSPR) optical characteristics using PC. The dotted line circles are LSPR peaks. (**a**) Experimentally obtained LSPR optical characteristics. (**b**) LSPR optical characteristics using simulation software.

The LSPR optical characteristics of the PC (thickness: 200 nm) are shown in Figure 5a. Using this PC configuration, LSPR peaks could be observed at approximately 530, 630, 720, and 780 nm. This LSPR peak wavelength was similar to that of the previously reported PC. To confirm LSPR excitation, the simulated spectrum of a PC in a similar configuration is shown in Figure 5b. By comparing the experimental and simulated spectra, we confirmed weak LSPR peaks at approximately 530, 630, 720, and 780 nm. The peak intensities differed from those in the simulation. By the SEM observation, similar sizes of gold pillar could be observed. However, the edges of gold pillars are not non-right angles. The edge angle will affect to the LSPR excitation efficiency. Hence, the differences of peak intensities could be observed. From these differences of peak intensities are expected the SERS efficiency. However, to confirm the usability of this PC for SERS application, this PC was thus used for SERS at 785 nm using commercially available portable Raman module.

**Figure 6:**
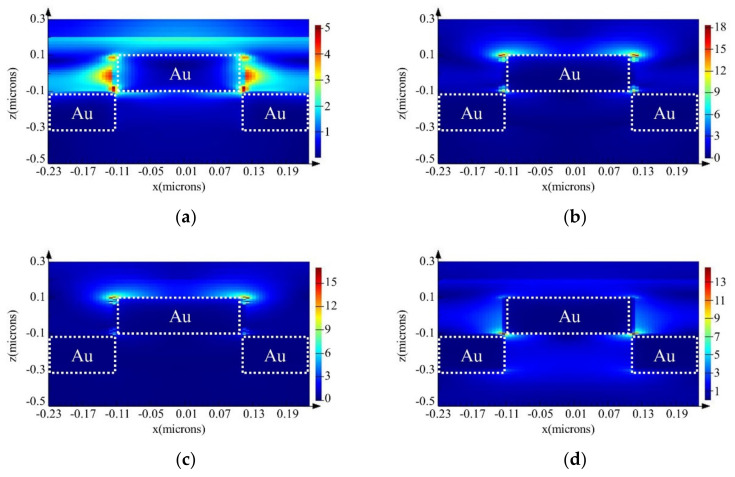
Electric field intensity distribution by LSPR on the gold nanostructures. (**a**) 532 nm, (**b**) 635 nm, (**c**) 650 nm, and (**d**) 785 nm.

The electric field intensity distribution on the gold nanostructure at different LSPR excitation wavelengths was analyzed by simulation. For SERS using the commercially available portable Raman module, the electric field was generated on the gold nanostructure surface at 785 nm. The electric field intensity distributions at different wavelengths are shown in Figure 6. From the LSPR optical characteristics, an electric field could be generated at each wavelength. According to the simulation analysis, the LSPR could be excited at 785 nm. However, the electric fields at each wavelength were extremely localized on the gold nanostructures at 785 nm. Hence, for SERS, target molecules should be absorbed on the area where the electric field was generated. If target molecules are positioned close to the surface of onto different areas of the gold nanostructures, a weak Raman signal is expected. In addition, the strong electric fields were formed at the edges of the gold nanostructures due to the edge effect [36]. However, from Figure 4, the sharp edges could not be observed. To obtain the more effective SERS active substrate, improvement of the deposition method of gold layer and precisely fabricated polymer mold are required. Furthermore, the distance between each gold nanostructure will be affected to the SERS efficiency. To obtain the highly effective SERS active substrate, closed distance is the better for SERS application. Based on these results, if the other wavelength were applied for SERS application, more effective SERS active substrate can be obtained.

### 3.3. SERS Using Template Stripping Method-Based PC by Detection of Phenobarbital

SERS using the PC was performed by depositing a 200 nm gold layer onto the template surface. We used the PC to successfully detect the Raman signal using insulin as a model case. To confirm the SERS effect, the Raman signal was monitored using a silicon substrate. As a result, the specific Raman signal of insulin could not be observed at the same concentration (data not shown). Hence, the detection of phenobarbital by SERS could be expected using this thickness of the PC.

**Figure 7:**
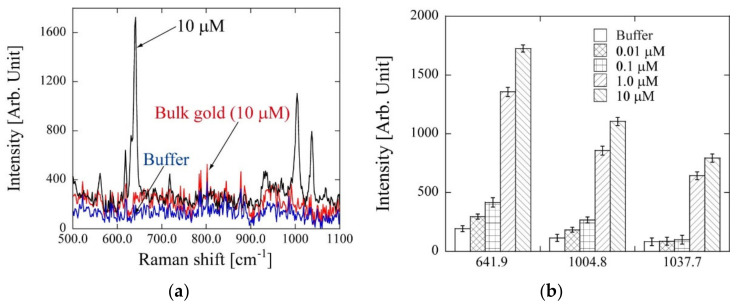
(**a**) Raman spectrum of phenobarbital using PC (black) and bulk gold substrate (red). In this study, PBS buffer was also introduced onto the PC surface as a blank (blue). Using PC, specific Raman signals could be observed at 641.9, 1004.8, and 1037.7 cm^−1^. (**b**) Concentration dependency for phenobarbital at 641.9, 1004.8, and 1037.7 cm^−1^.

Raman spectra at different concentrations of phenobarbital obtained using the PC are shown in Figure 7a. Using this PC, Raman signals could be observed at 641.9, 1004.8, and 1037.7 cm^−1^. Based on the chemical structure of phenobarbital, these signals were attributed to the phenyl group and sodium salt [37]. To compare the SERS effect, a bulk gold substrate was used for a similar experiment. We could not obtain the SERS effect for 10 µM of phenobarbital using the bulk (flat) gold substrate (Figure 7a). By introducing different concentrations of the phenobarbital solution, specific Raman signals that originated from phenobarbital could be observed at 1.0 µM at 641.9, 1004.8, and 1037.7 cm^−1^ (Figure 7b).

The previously reported detectable concentration using SERS was micromolar order [38] or higher [39]. This PC is expected to perform well in SERS. The simulation analysis result proved that phenobarbital needs to be adsorbed onto the localized area of gold nanostructures that generated the electric field by LSPR. The SERS result showed that phenobarbital was preferentially absorbed owing to the capillary force.

## 4. Conclusions

The PC was successfully fabricated (for SERS application) rapidly and cost-effectively using the template stripping method. We could also detect phenobarbital with high sensitivity using this PC and commercially available portable Raman module. These results prove that this PC could be applied in several fields, such as life sciences and pharmaceutical sciences. From the simulation analysis, this configuration of the PC showed SERS activity at different wavelengths. The sensitivity of this PC for SERS can be improved by using the appropriate design (size and shape) and material of the PC and controlling the optimized wavelength of the light source.
